# Yeast PIC-Mediator structure with RNA polymerase II C-terminal domain

**DOI:** 10.1073/pnas.2220542120

**Published:** 2023-04-04

**Authors:** Sandra Schilbach, Haibo Wang, Christian Dienemann, Patrick Cramer

**Affiliations:** ^a^Department of Molecular Biology, Max Planck Institute for Multidisciplinary Sciences, Göttingen 37077, Germany

**Keywords:** gene transcription, RNA polymerase II initiation, Mediator, RNA polymerase II C-terminal domain, cryo-electron microscopy

## Abstract

For transcription initiation, RNA polymerase II (Pol II) forms a preinitiation complex (PIC) that associates with the general coactivator Mediator. Whereas atomic models of the human PIC-Mediator structure have been reported, structures for its yeast counterpart remain incomplete. Here, we present an atomic model for the yeast PIC with core Mediator, including the Mediator middle module that was previously poorly resolved and including subunit Med1 that was previously lacking. We observe three peptide regions containing eleven of the 26 heptapeptide repeats of the flexible C-terminal repeat domain (CTD) of Pol II. Two of these CTD regions bind between the Mediator head and middle modules and form defined CTD–Mediator interactions. CTD peptide 1 binds between the Med6 shoulder and Med31 knob domains, whereas CTD peptide 2 forms additional contacts with Med4. The third CTD region (peptide 3) binds in the Mediator cradle and associates with the Mediator hook. Comparisons with the human PIC-Mediator structure show that the central region in peptide 1 is similar and forms conserved contacts with Mediator, whereas peptides 2 and 3 exhibit distinct structures and Mediator interactions.

## Significance

At gene promoters, RNA polymerase II (Pol II) assembles into a preinitiation complex (PIC) which includes the coactivator Mediator. Here, we describe the PIC-Mediator complex from yeast at unprecedented resolution and provide a reference structure for initiation complexes. We obtain insights into the atomic details of Mediator and its interaction with Pol II. Moreover, we observe three Mediator-bound fragments of the C-terminal domain (CTD) of Pol II which stabilize Mediator conformation within the PIC and significantly extend prior information from the human system. These peptide regions correspond to almost 50% of the yeast CTD and thus the minimal length required for cell viability. Their location and conformation are in part extremely conserved, highlighting their central role in transcription.

For the initiation of transcription, RNA polymerase II (Pol II) assembles with the general transcription factors into a preinitiation complex (PIC) on promoters of protein-coding genes (1–3). Recent studies provided various PIC structures from the yeast *Saccharomyces cerevisiae* (4–8) and the human system (9–14). Despite these advances, only a partial model of the yeast PIC-Mediator complex is currently available and only the Mediator head module has been described at high resolution (5–8). Also, the Pol II C-terminal repeat domain (CTD) has been localized within the human PIC-Mediator complex, but not within its yeast counterpart. Moreover, the resolution for the Mediator-bound CTD regions remains limited in the human structure. Therefore, a high-resolution yeast PIC-Mediator structure that also elucidates the path of the bound CTD remained an important goal in the transcription field.

Here, we report the high-resolution structure of the yeast PIC-core Mediator complex that we could stabilize by addition of the +1 nucleosome. We reveal two stretches of the CTD bound between the Mediator head and middle modules, as well as one CTD stretch at the Mediator hook domain. Comparison with the available human structures reveals conserved and species-specific CTD features and Mediator–CTD interactions.

## Results

### Structure Determination.

Despite enormous efforts over the years, there is currently no protocol to obtain well-defined, complete recombinant yeast Mediator for structural studies. We therefore prepared a 16-subunit core Mediator complex (cMed) and bound it to a preassembled yeast PIC (5, 7) that was extended by a +1 nucleosome as recently reported (15). We obtained a stable PIC-cMed-nucleosome complex that was suited for structural analysis (*Materials and Methods*). Single-particle cryo-electron microscopy (cryo-EM) analysis led to reconstructions of the PIC-cMed complex lacking the nucleosome and the PIC-cMed-nucleosome complex at 3.0 Å and 3.6 Å resolution, respectively (*Materials and Methods* and *SI Appendix*, Figs. S1 and S2). In the nucleosome-containing PIC-cMed structure, the nucleosome was oriented as observed before in a PIC-nucleosome complex lacking cMed (15) and did not contact Mediator (*SI Appendix,* Fig. S3). Therefore, we did not obtain additional insights into the interactions of the nucleosome with the PIC compared to what was previously described (15), and do not describe the nucleosome further here.

### Model Building.

To build the PIC-cMed structure, we first fitted our previously reported atomic PIC structure into the cryo-EM map of the PIC-cMed complex (4). The PIC was adjusted using a focused map at 2.9 Å resolution. Focused refinement of cMed then led to a local resolution of 3.3 Å (*SI Appendix,* Fig. S1) and enabled us to build and refine an atomic model of cMed that was based on our previous partial model (5). We then used AlphaFold2 to obtain a highly reliable model of Mediator subunit Med1 (16). This Med1 model was placed unambiguously into an unassigned density next to the Mediator plank domain located near the Pol II foot region (*Materials and Methods*).

Further focused refinement yielded a high-resolution reconstruction (3.1 Å) of the central cMed region including the shoulder, knob, connector, and hook domains and permitted the localization of additional densities corresponding to parts of the Pol II CTD. Those densities were mainly observed on the central Mediator surface, near the location at which Pol II CTD peptide regions have been observed in human PIC-Med structures (11, 14). The obtained high resolution of the cryo-EM map enabled us to build atomic models for three CTD regions which we refer to as “CTD peptide 1”, “CTD peptide 2”, and “CTD peptide 3”. In conclusion, we obtained an atomic model of the yeast PIC-cMed complex that includes the Mediator middle module, Med1, and three Mediator-bound CTD regions and shows very good stereochemistry (*SI Appendix*, Fig. S2 and Table S1).

### High-Resolution Yeast PIC-Mediator Structure.

**Fig. 1.**
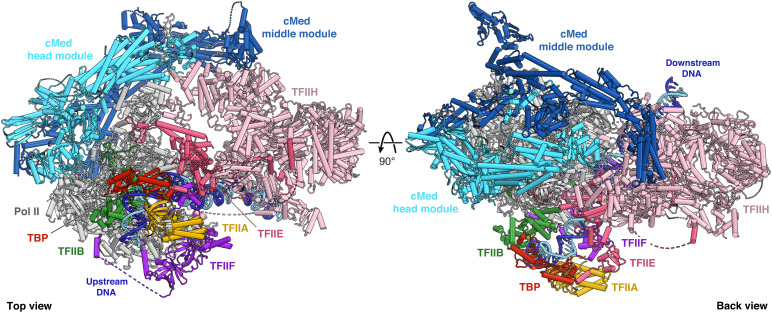
Structure of the yeast PIC-cMed complex. Two views of the PIC-cMed complex structure depicted in ribbon representation. Major submodules are distinguished by color. The DNA template and nontemplate strands are shown in dark and light blue, respectively. Dashed lines represent flexible linkers.

**Fig. 2.**
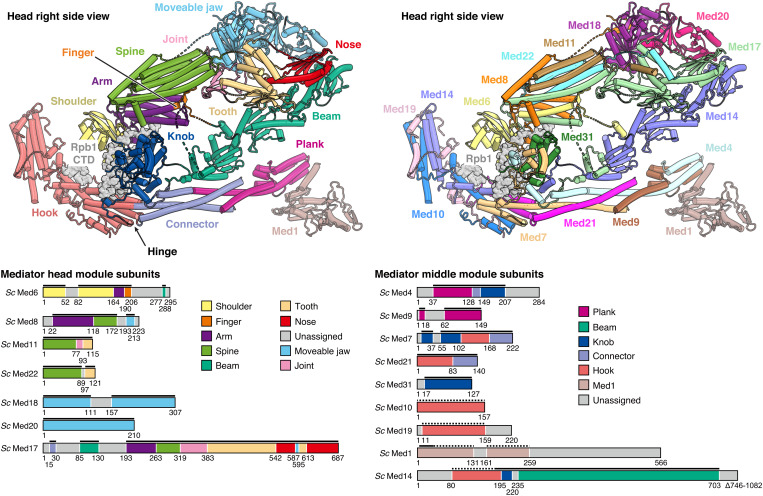
PIC-bound Mediator structure. Structure of yeast cMed in the PIC-bound state. Domain (*Top Left*) and subunit (*Top Right*) architecture of the improved cMed model. The Rpb1 CTD is represented as a gray surface. cMed domain colors were adapted from the *Schizosaccharomyces pombe* cMed structure (17). The same color code is applied for schematic depiction of the cMed subunit domain organization and permits distinction of subunit regions contributing to respective cMed modules (*Bottom*). Residue numbers demarcate domain boundaries. Solid black bars indicate regions modeled at high resolution with side-chain information. Dashed black bars represent model sections that were predicted by AlphaFold 2 (16, 18) and adapted into the experimental cryo-EM density by flexible fitting (19).

The refined 46-subunit PIC-cMed structure containing the CTD provides an improved reference structure for yeast transcription initiation complexes (Fig. 1). Whereas the PIC structure is virtually identical to our previously published atomic models (4, 15), the atomic model of the cMed structure now includes also the middle module, for which a detailed structure was only available in the free state (17). Our structure contains extended models for all middle module subunits, as well as more complete models for Med17 and the previously lacking subunit Med1 (Fig. 2).

The structure provides a detailed view of the interaction between the Mediator plank and the Pol II foot domain and Rpb8, which is mediated through a hydrophobic and a polar interface (*SI Appendix,* Fig. S4). The presence of Med1 appears to stabilize the contacts between Pol II and cMed plank subunits Med4 and Med9 (*SI Appendix,* Fig. S5). Comparison of the PIC-bound cMed structure with the free cMed crystal structure shows that the structure is largely unaltered by PIC binding, except that the middle module moved with respect to the head, leading to a more extended conformation as described before (5). In contrast to the human system (11, 12, 14), the TFIIH kinase module remains flexible in the yeast structure also in the presence of Mediator (*SI Appendix,* Fig. S1) and was therefore not included in the final model.

### CTD Regions Bridge the Mediator Head and Middle Modules.

**Fig. 3.**
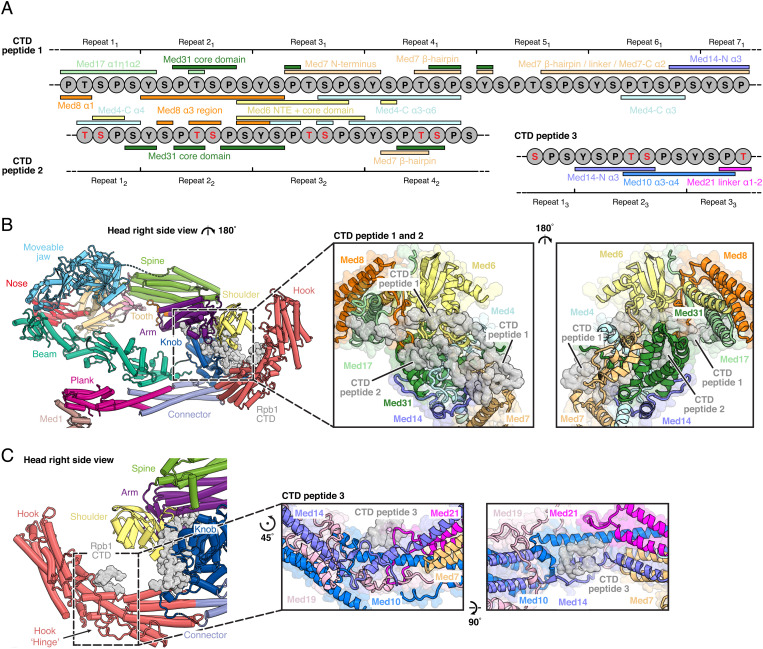
Overview of CTD–Mediator interactions. (*A*) Schematic representation of the “bridging” CTD peptides 1 and 2, as well as CTD peptide 3, and their interaction partners in the Mediator head and middle modules. A total of 11 complete CTD repeats establishes an extensive interaction network with nine subunits (Med4, Med6, Med7, Med8, Med10, Med14, Med17, Med21, and Med31) in both cMed modules. Red letters in CTD peptides 2 and 3 illustrate ambiguous directionality of the CTD within these segments, resulting in undefined connectivity between the CTD peptides. The directionality of CTD peptide 1 could be determined with certainty in the cryo-EM density. Domains and regions in cMed are labeled based on previous nomenclature (17, 20). Color code is as in Fig. 2. Residues contributing to interface formation were identified using the PISA web server (21). (*B*) Two Pol II CTD segments wrap tightly around the Mediator arm, knob, and shoulder domains and extend toward the hinge and hook regions. Overview of the cMed complex viewed from the back. The Rpb1 CTD is represented as a surface in gray. The path of the CTD is magnified and depicted from opposing sides, highlighting its intimate interface with Mediator. Interacting cMed subunits are distinguished by color. The distinct CTD peptides are demarcated. Color codes are as in Fig. 2. (*C*) Position and trajectory of CTD peptide 3. Illustration of the shortest observed CTD peptide and its interface with the Mediator hook domain. The Rpb1 CTD is represented as a surface in gray and its path is depicted from two views. The fragment adopts a hairpin-like structure and folds onto the surface of the central hinge-like region of the Mediator hook domain. Domains and regions in cMed are labeled based on previous nomenclature (17, 20). Color codes are as in Fig. 2.

The yeast CTD contains 26 heptapeptide repeats of the consensus sequence Y^1^S^2^P^3^T^4^S^5^P^6^S^7^. The largest of the three CTD regions observed here (CTD peptide 1) comprises six complete heptapeptide repeats, whereas the other regions (CTD peptide 2 and CTD peptide 3) comprise three and two repeats, respectively (Fig. 3*A*). Thus, our structure contains 11 out of the 26 repeats that cover a total of 82 residues, i.e., 45% of the entire CTD. CTD peptides 1 and 2 protrude from beneath the Mediator arm and create an interface between the shoulder and knob domains, which reside in the Mediator head and middle modules, respectively (Fig. 3*B* and *SI Appendix,* Fig. S6). Therefore, the CTD forms a bridge between the two modules of core Mediator, apparently stabilizing the mobile Mediator middle module. CTD peptide 3 binds the Mediator hook domain (Fig. 3*C*).

Although details differ, the overall location of the central region in CTD peptide 1 resembles that of a soaked peptide in a previous crystal structure of the isolated *S. cerevisiae* Mediator head module (22). The location of CTD peptide 1 also roughly corresponds to the location of a CTD fragment in a medium-resolution cryo-EM structure of the *S. pombe* Mediator-Pol II complex (23). In contrast to these previous results, however, the density for CTD peptide 1 obtained here is highly detailed and more extended. The density allowed us to assign side chains and to unambiguously define the directionality and register of CTD peptide 1. In addition to previous studies in yeast, we observed two additional CTD regions, peptides 2 and 3. In the following paragraphs, we first describe the interactions of all three CTD regions in detail and then compare our observations with findings from the human system.

### CTD–Mediator Interactions.

**Fig. 4.**
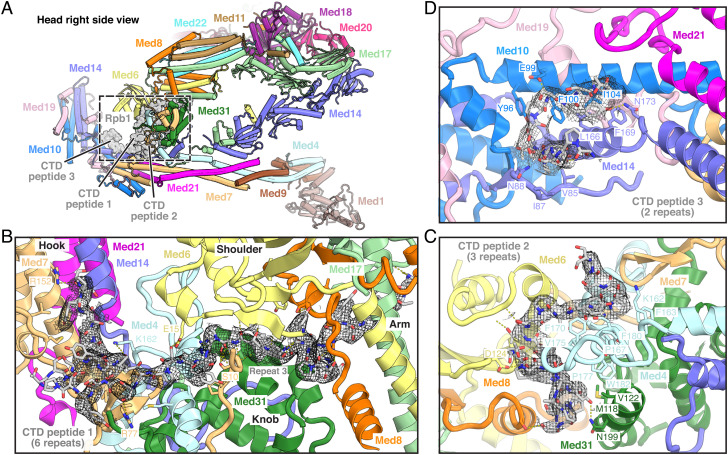
Details of CTD–Mediator interactions. (*A*) Overview of location and trajectory of the three Pol II CTD segments bound to cMed. The Rpb1 CTD is represented as a surface in gray. Color code is as in Fig. 2. (*B*) Detailed view of the longest observed Pol II CTD segment (CTD peptide 1). CTD peptide 1 extends from the cMed arm to the shoulder and knob domains and intimately contacts the cMed head and middle modules, thereby serving as a “molecular glue” to stabilize the overall cMed conformation. Key residues in cMed that are involved in contacts with the Pol II CTD are denoted. CTD peptide 1 and the corresponding cryo-EM density are illustrated as sticks and as a gray mesh, respectively. (*C*) Detailed view of the second Pol II CTD segment (CTD peptide 2). Multiple subunits in the cMed head and middle modules interface with the CTD peptide 2 trajectory. Key residues in cMed that are involved in contacts with the Pol II CTD are denoted. The CTD peptide and the corresponding cryo-EM density are illustrated as sticks and as a gray mesh, respectively. (*D*) Detailed view of the shortest observed Pol II CTD segment (CTD peptide 3). CTD peptide 3 is wedged into an internal hinge in the cMed hook domain. Key residues in cMed that are involved in contacts with the Pol II CTD are denoted. The CTD peptide and the corresponding cryo-EM density are illustrated as sticks and as a gray mesh, respectively. Panels *B–D* are viewed from the inner side of the cMed cradle toward the respective CTD peptides.

The two CTD peptide regions 1 and 2 are adjacently located in between the Mediator head and middle modules (Fig. 4*A*). The first heptapeptide repeat of CTD peptide 1 binds to a cavity created by Med8 and Med17, whereas the second repeat forms a short β-strand that contacts Med8 (Fig. 4*B*). The third repeat forms a wedge between Med6, the Mediator knob formed by subunit Med31, the N-terminal region of Med7, and the C-terminal region of Med4. Finally, repeats 4-6 of CTD peptide 1 interact with the Med7 β-hairpin and C-terminal extension (Fig. 4*B*).

CTD peptide 2 further contributes to connect the Mediator head and middle modules (Fig. 4*C*). One half of CTD peptide 2 forms a loop located between the Mediator head module subunits Med6 and Med8 on one side and the Mediator middle module subunits Med4 and Med31 on the other side. The other half of CTD peptide 2 meanders along Med6 and reaches the Med4 C-terminal region and Med7 β-hairpin to add to the CTD-mediated contacts between the Mediator head and middle modules (Fig. 4*C*).

CTD peptide 3 is located in the Mediator cradle (7) at a central hinge within the hook domain. It stabilizes the hook conformation through interactions with subunits Med10, Med14, and Med21 (Fig. 4*D*). The observed location of CTD peptide 3 agrees well with previously reported cross-links to Med19 lysine residues 56, 59, and 62 (7) (*SI Appendix,* Fig. S6). Densities corresponding to CTD peptides 2 and 3 had not been reported in any structural analyses of yeast PIC-Mediator complexes before.

### Comparison with Human CTD Fragments.

**Fig. 5.**
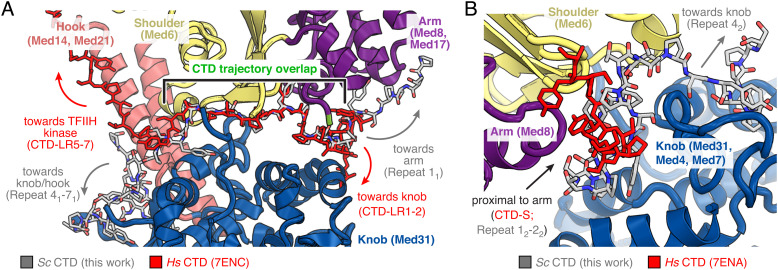
Comparison of Mediator-bound yeast and human CTD. (*A*) Comparison of yeast CTD peptide 1 to the corresponding Mediator-bound human CTD peptide reveals a conserved central path. Structures of human (14) and yeast complexes were aligned on Mediator head module subunit Med6 (human MED6). For clarity, only the yeast structure is depicted in ribbon representation. The CTD trajectories in the yeast and human complexes are almost identical in direct proximity of the cMed head–middle module interface (repeat 2_1_-3_1_ and CTD-LR3-4, respectively) but significantly diverge for the adjacent N- and C-terminal repeats. CTD fragments are shown as sticks in gray (yeast) and red (human). Color code of the cMed cartoon representation is as in Fig. 2. View adapted from Fig. 4*B*. (*B*) CTD peptide 2 is located at similar positions in the yeast and human (14) complexes but adopts distinct conformations. Structures of human and yeast complexes were aligned on Mediator head module subunit Med6 (human MED6) as in panel *A*. For clarity, only the yeast structure is depicted in ribbon representation. Conformational differences in CTD peptide 2 may be a product of evolutionary divergence in CTD affinity to the surface of Mediator. Also, a longer interacting CTD segment was observed in the yeast structure. CTD fragments are shown as sticks in gray (yeast) and red (human). Color code of cMed cartoon representation is as in Fig. 2. View adapted from Fig. 4*C*.

Comparison of our structure with the best-resolved human PIC-Mediator structure (14) shows that parts of the Mediator–CTD interactions are conserved. In particular, the central region of yeast CTD peptide 1 follows a path that is similar to the path taken by two repeats of the human CTD segment CTD-L that are referred to as CTD-LR3 and CTD-LR4 (Fig. 5*A*). However, the N-terminal region of the CTD-L fragment (CTD-LR1-2) does not interact with the Mediator arm as observed for the yeast CTD peptide 1. Instead, it extends toward the Mediator middle module subunit Med31 (human MED31). Another difference relates to the path of the human CTD repeats CTD-LR5-7 after they exit the groove formed between the Mediator knob and shoulder domains. Instead of directly projecting toward the tip of the Mediator hook and the TFIIH kinase module, yeast CTD peptide 1 further winds around the Mediator knob before contacting only the proximal region of the hook domain (Fig. 5*A*).

The density observed for yeast CTD peptide 2 partially overlaps with the human CTD fragment CTD-S, but the position and trajectory differ significantly between both species (Fig. 5*B*). In comparison to the human fragment, yeast CTD peptide 2 is also extended and spans three heptapeptide repeats that permit a more intimate interaction with the Mediator knob domain. An equivalent of yeast CTD peptide 3 has not been reported for any human initiation complexes to date. It therefore remains to be seen whether this is a species-specific difference or whether future work on the human system will reveal a counterpart to yeast CTD peptide 3. In summary, the overall location of the CTD between the Mediator head and middle modules is conserved between yeast and human systems, as are CTD-Mediator contacts formed by the central part of CTD peptide 1, but major portions of the CTD regions differ between yeast and human with respect to their structure and Mediator interactions.

## Discussion

Here, we extend our structural understanding of the yeast transcription initiation machinery. We expand the atomic model of the PIC-cMed complex to the Mediator middle module, Med1, and three regions of the Pol II CTD that were previously not available or only resolved at very low resolution. Comparison of our structures with available human models (11, 12, 14) shows similarities, reflecting the overall high degree of conservation of the transcription initiation complexes between eukaryotic species. The locations of the two CTD peptide regions that were observed in both the yeast and the human systems are similar, including a strong conservation of a central CTD stretch at the interface between the Mediator head and middle modules. This suggests a pivotal role of the CTD in stabilizing a closed Mediator conformation to prime the assembled PIC for the start of transcription, in line with previous observations that implied the CTD–Mediator interaction as an important functional association (24).

However, the comparison also revealed differences in the molecular architectures of the yeast and human complexes. First, the TFIIH kinase module in the yeast complex remains flexibly associated, whereas the human module adopts a more defined position (12, 14). Second, the core Mediator structures are highly conserved, except for the previously observed different relative orientation of the Mediator middle module with respect to the head module (12). Third, peripheral CTD regions differ between the yeast and human complexes and CTD peptide 3 appears to be unique to the yeast system.

Finally, it is striking that the number of eleven observed CTD repeats in our structure corresponds to the number of CTD repeats that is minimally required for yeast cell viability (25, 26) and overlaps with corresponding CTD fragments identified in a functional screen in terms of range, relative location, and potential connectivity (27). In contrast to the yeast CTD, which contains 26 repeats, the human CTD is twice the length and comprises 52 repeats. Whereas the yeast CTD contains only very few degenerate heptapeptide repeats, about 50% of the human CTD deviate from the consensus sequence. Those degenerate repeats may not bind as efficiently and interchangeably to the surface of Mediator and instead may remain flexible, which could explain some of the differences observed between the yeast and human structures. We suggest that the 11 repeats of the yeast CTD shown here represent the minimal, evolutionarily conserved and essential part of the CTD required for its basal role in transcription, and that the CTD has been extended during evolution to enable additional functions such as the coordination of co-transcriptional processes (28) and efficient transcriptional regulation (29).

## Materials and Methods

### Protein Preparation.

Preparation of *S. cerevisiae* Pol II, TBP, TFIIA, TFIIB, TFIIE, TFIIF, TFIIH, and 16-subunit cMed was performed as described (5, 7, 30). Unless specifically stated, all purification procedures were performed at 4 °C.

### Cryo-EM Sample Preparation and Data Collection.

The PIC-cMed-nucleosome complex was prepared according to a protocol adapted from the previously reported assembly scheme for PIC-cMed (5, 30) and PIC-nucleosome (15). The DNA scaffold containing the modified *His4* promoter and the Widom-601 sequence (underlined) were synthesized by Integrated DNA Technologies (IDT), amplified, and purified as described (15) with the template strand sequence: 5​′-A​GCA​CGC​TGT​GTA​TAT​AAT​AGC​TAT​GGA​ACG​TTC​GAT​TCA​CCT​CCG​ATG​TGT​GTT​GTA​CAT​ACA​TAA​AAA​TAT​CAT​AGC​TCT​TCT​GCG​CTG​TGT​TG​GTC​GTA​GAC​AGC​TCT​AGC​ACC​GCT​TAA​ACG​CAC​GTA​CGC​GCT​GTC​CCC​CGC​GTT​TTA​ACC​GCC​AAGGGGATTACTCCCTAGTCTCCAGGCACGTGTCAGATATATACATCGAT-3′. Nucleosomes were assembled by salt gradient dialysis as described (15).

To form the complex, TFIIA, TFIIB, TBP, and nucleosome-containing template were incubated for 5 min. Preincubated Pol II—TFIIF was added to obtain a Pol II/IIA-IIB-TBP-IIF-nucleosome complex. TFIIE, TFIIH, and cMed were combined, incubated for 10 min, and then added to the sample. The complete assembly mix was incubated for another 120 min while gently shaking at 400 rpm. The sample was purified by sucrose gradient ultracentrifugation following the GraFix protocol (31). The gradient was prepared using a 15% sucrose solution [15% (w/v) sucrose, 100 mM KCl, 25 mM K·4-(2-hydroxyethyl)-1-piperazineethanesulfonic acid) (HEPES) pH 7.5, 2 mM MgCl_2_, 2.5% glycerol (v/v), 1 mM tris(2-carboxyethyl)phosphine (TCEP)] and a 40% sucrose solution [40% (w/v) sucrose, 100 mM KCl, 25 mM K·HEPES pH 7.5, 2 mM MgCl_2_, 2.5% glycerol (v/v), 1 mM TCEP, 0.1% (v/v) glutaraldehyde] with a BioComp Gradient Master 108 (BioComp Instruments). Centrifugation was performed at 175,000 × g for 16 h at 4 °C. Subsequently, 200 µL fractions were collected, quenched with a pH-adjusted mix of 40 mM aspartate and 10 mM lysine (10 min, 4 °C), and analyzed by native PAGE. Fractions containing cross-linked complex were dialyzed in Slide-A-Lyzer MINI Dialysis Devices (2 mL, 20,000 MWCO) (Thermo Fisher) for 8 h against dialysis buffer (100 mM KCl, 25 mM K·HEPES pH 7.5, 2 mM MgCl_2_, 1 mM TCEP) to remove sucrose and glycerol before being used for cryo-EM grid preparation. Four microliters of the PIC-cMed-nucleosome sample was applied to UltrAuFoil 2/2 grids (Quantifoil) that had been glow-discharged immediately prior. Grids were blotted for 3 s and vitrified by plunging into liquid ethane with a Vitrobot Mark IV (Thermo Fischer) operated at 4 °C and 100% humidity.

Data collection was performed automatically with SerialEM (32) on a Titan Krios G2 transmission electron microscope (Thermo Fisher) operated in energy-filtered transmission electron microscopy (EFTEM) mode at 300 kV and a Quantum LS energy filter (Gatan) with a slit width of 20 eV. Cryo-EM data were obtained on a K3 direct electron detector (Gatan) with a calibrated pixel size of 1.05 Å (nominal magnification of 81,000×) and a dose of approximately 41.3 electrons/Å^2^ fractionated over 40 frames. A defocus range from −0.8 to −2.0 μm was applied.

### Cryo-EM Image Processing.

Movie frames of 33,548 micrographs were aligned, contrast transfer function (CTF)-estimated, motion-corrected, and dose-weighted using Warp (33). Particles were automatically picked with Warp, resulting in 5,381,599 initial particles. Subsequent steps of image processing were performed with RELION-3 (version 3.1.0) (34–36) unless stated otherwise. three-dimensional (3D) classifications and refinements are referred to as “focused” if local masking was applied. Refinements were conducted with fully independent half-sets of the data (“gold standard”), and map resolution was determined based on the Fourier shell correlation = 0.143 criterion (37). Postprocessing was performed with user-provided or automatic B-factor determination and sharpening in RELION or with the “Noise2Map” algorithm in the Warp package. Local resolution was estimated with the built-in tool in RELION without B-factor sharpening. Masks were generated with UCSF Chimera (38) and RELION.

Particle coordinates were imported into RELION and extracted with a binning factor of 2 (pixel size 2.1 Å/pixel) and a box size of 240 pixels. Initial particle cleanup was performed by alternating rounds of reference-free two-dimensional (2D)- and template-guided global 3D classification, using cryoSPARC (39). A resulting set of 2,717,155 particles with well-defined cPIC features was reextracted without binning (pixel size 1.05 Å/pixel), refined in 3D, and subjected to two rounds of CTF refinement and Bayesian polishing in RELION to correct for beam-induced motion. Next, particles containing either well-defined cMed or well-defined TFIIH were identified in separate approaches, yielding 741,377 cMed particles and 685,473 TFIIH particles which were refined with local angular sampling to a global resolution of 3.2 Å and 3.3 Å, respectively. Particles containing well-defined cMed were further curated by focused 3D classification with a loose mask encompassing the cMed hook domain, which revealed two classes with a strong signal for the complete cMed hook domain. These particles were merged (194,910 particles) and locally refined with a mask around cMed hook domain, yielding a 3.3 Å reconstruction. Particles containing well-defined TFIIH were further curated by focused 3D classification with a loose mask encompassing the nucleosome, which revealed three classes (together 173,733 particles) with a strong signal for the +1 nucleosome. These particles were then locally refined with a mask around the +1 nucleosome, yielding a 3.4 Å reconstruction.

Particles containing both good cMed and TFIIH were selected (186,599 particles) and refined to yield a 3D reconstruction with global resolution at 3.0 Å. Each component of PIC-cMed complex in the final reconstruction was improved by focused 3D refinement. Particles containing good cMed, TFIIH, and nucleosome (50,715 particles) were selected and refined to yield a 3D reconstruction with global resolution at 3.6 Å. Each component of PIC-cMed-nucleosome complex in the final reconstruction was improved by focused 3D refinement. Ultimately, the focused maps were combined with Warp (33) to generate a composite map for model building and refinement.

### Model Building and Refinement.

Model building for each component of the PIC-cMed-nucleosome complex was performed in the composite cryo-EM map. Models were rigidly docked into densities in UCSF Chimera (38), whereas COOT (40) was utilized for accurate domain fitting as well as manual model modification and de-novo model building. Real space refinement was conducted with the PHENIX suite (41, 42). Figures were created with PyMOL (version 2.4), UCSF Chimera, and UCSF ChimeraX (43).

The coordinates of the high-resolution structure of the closed yeast PIC (PDB accession code 7O72) (4) were used as a starting model. Individual chains of the model were rigidly docked into the density and manually adjusted and adapted in COOT to improve the fit where necessary. A final closed PIC model with good stereochemistry was then obtained by iterative rounds of manual model adjustment in COOT and real space refinement.

The high-resolution structure of cMed was generated based on the previously published yeast PIC-cMed model (PDB accession code 5OQM) (5). Coordinates of cPIC and TFIIH subunits were removed and the remaining chains were individually docked into the density. Poorly fitting regions were adapted in COOT. The model was extended de-novo to include previously unobserved segments such as the N termini of Med9 or Med17 or the central part of Med7. Side chains, which had not been modeled previously, were added to the respective residues and manually adjusted in COOT. Med1 was predicted using AlphaFold 2 (16, 18), stripped of long unstructured extensions, rigidly docked into the cryo-EM density, and flexibly fitted with Namdinator (19). The N terminus of Med1, which was observed at side-chain resolution, was manually optimized in COOT. The C terminus of Med1, for which cryo-EM density was lacking, was truncated. Three Pol II CTD peptides spanning over a total of eleven YSPTSPS-repeats were modeled de-novo. Due to its repetitive nature, it was difficult to assign register to those CTD fragments. Thus, the segments were numbered according to the length of the observed repeat fragments, with the longest fragment becoming #1 and the shortest becoming #3. An initial model of the cMed hook domain was determined with AlphaFold 2 (16, 18). For multimeric structure prediction, sequences of cMed subunits Med7 (110 to 170), Med10 (residues 1 to 157), Med14 (residues 80 to 195), Med19 (residues 11 to 159), and Med21 (residues 1 to 85) were provided. The obtained hook model was flexibly fitted into the focused “hook side” cryo-EM map with Namdinator and manually adjusted in COOT. A final cMed model was then obtained by iterative rounds of manual model adjustment in COOT and real space refinement in PHENIX and displayed good stereochemistry as assessed by MolProbity (44).

## Supplementary Material

Appendix 01 (PDF)

## Data Availability

Structural models, cryo-EM densities data have been deposited in PDB, EMDB (EMD-16610, EMD-16611, PDB 8CEN, PDB 8CEO).
