# Supplementary material for: Yeast PIC-Mediator structure with RNA polymerase II C-terminal domain
Source: Proc Natl Acad Sci U S A. 2023 Apr 4;120(15):e2220542120. doi: 10.1073/pnas.2220542120 (PMC10104585; doi:10.1073/pnas.2220542120)
Supplement: Supplementary file 1 — Appendix 01 (PDF) [file pnas.2220542120.sapp.pdf]

PNAS

Supporting Information for
Yeast PIC-Mediator structure with RNA polymerase II C-terminal domain.

Sandra Schilbach, Haibo Wang, Christian Dienemann and Patrick Cramer*

* Corresponding author.

Email: patrick.cramer@mpinat.mpg.de

This PDF file includes:

Figures S1 to S6
Table S1
SI References

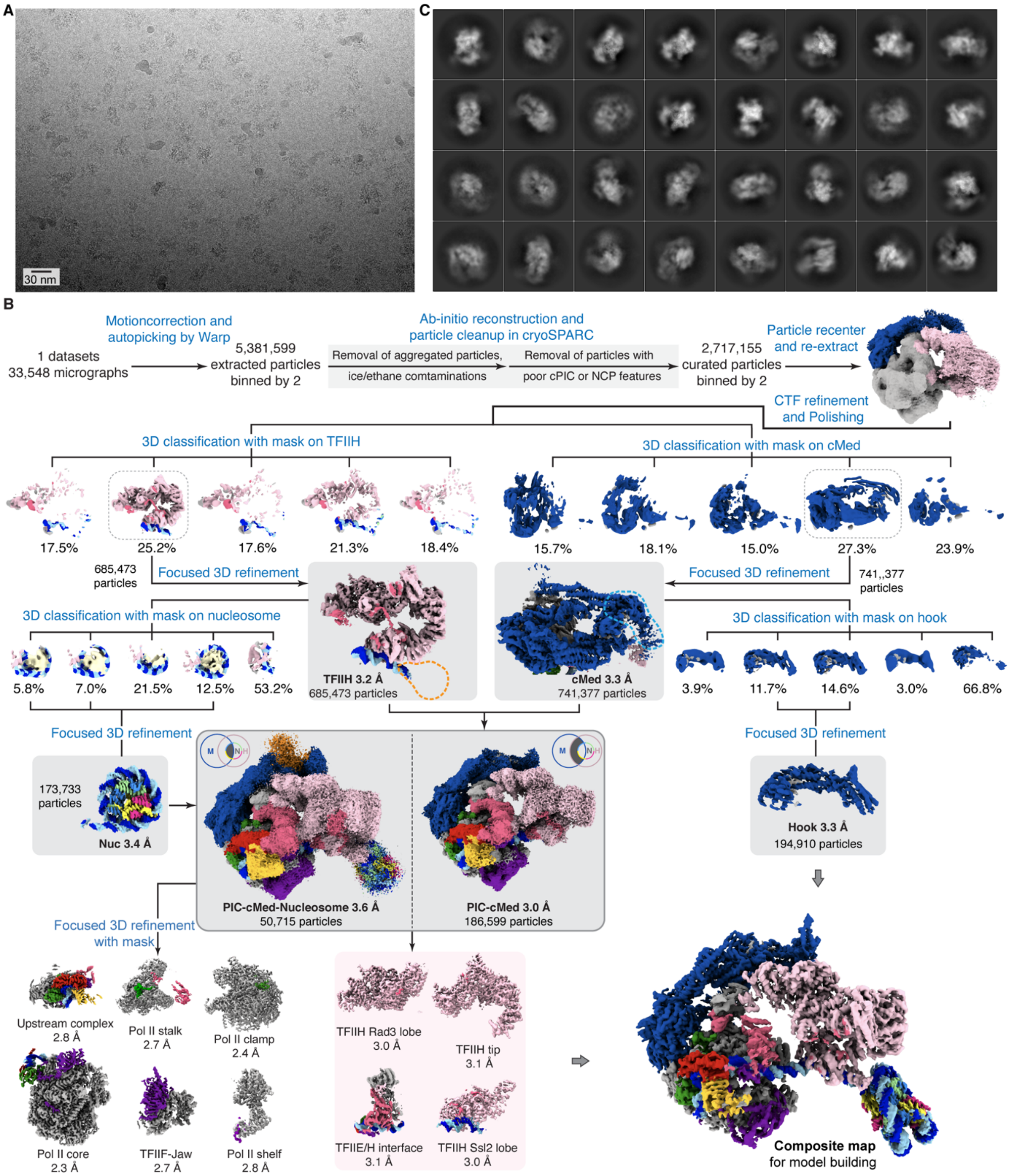

Figure S1. Cryo-EM data collection and processing of PIC-cMed-nucleosome complex. *(A)* Representative cryo-EM micrograph. A scale bar is provided. *(B)* Particle sorting and classification tree. The scheme illustrates how high-resolution cryo-EM reconstructions of PIC, cMed and nucleosome were obtained from the original cryo-EM dataset by multiple rounds of classification and refinement. Masks applied during classification and refinement are depicted as dotted outlines or specified in the accompanying text in blue. Complex subunits and submodules are highlighted according to the color code in Fig. 1 and cMed is colored in dark blue. During post-processing, maps were sharpened and filtered in RELION (1). Final reconstructions that were deposited to the

EMDB are represented on a light grey background and outlined in black. Global resolution estimates are provided. *(C)* Representative 2D class averages of final sorted particles. In addition to well-defined PIC and cMed regions, weak additional signal corresponding to the nucleosome is observed.

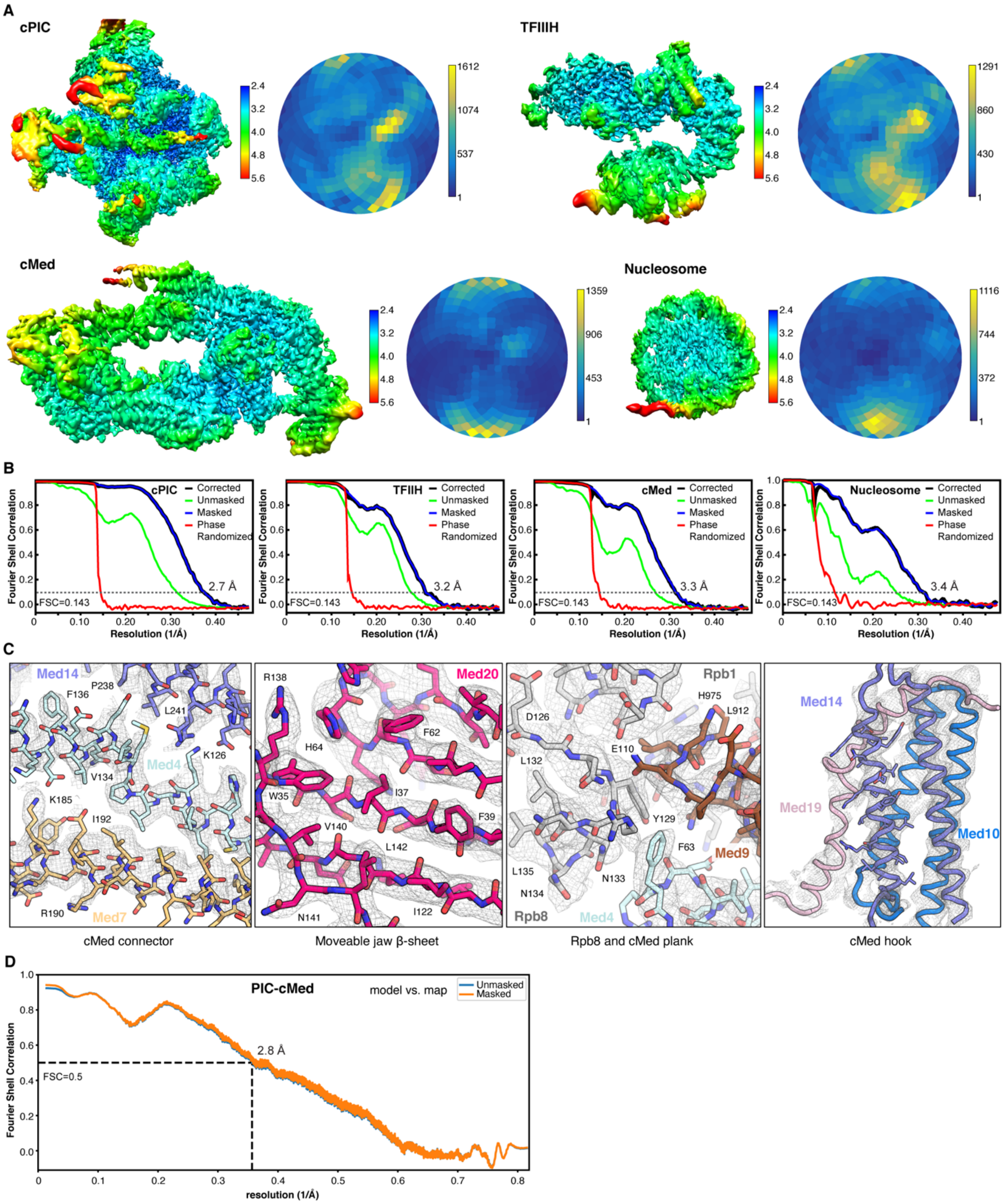

Figure S2. Quality of cryo-EM reconstructions and structural models. *(A)* Local resolution distribution. Final reconstructions of the closed cPIC, cMed, TFIIH and nucleosome complexes are depicted and colored according to local resolution [Å] as indicated. Angular distribution plots for particles included in the final reconstructions were determined with the Warp toolbox (2) and particle populations at given angular orientations are indicated by shading in color. *(B)* Solvent-corrected Fourier shell correlations (FSCs) between half maps of the final reconstructions are indicated and the resolution reported at the FSC=0.143 cut-off criterion (dashed line) is provided. *(C)*

Representative regions of the final cryo-EM maps overlaid with their corresponding refined structural models. Distinct sections of the improved cMed structure are depicted and illustrate a wide resolution range in which modeling was performed using various approaches (*Methods*). Cryo-EM densities are depicted as grey mesh and selected residues in the model are denoted for orientation. Color code as in Fig. 2. *(D)* Model-to-map Fourier shell correlation. Correlation curves were calculated based on refined atomic coordinates of the model of the PIC-cMed complex and indicate their fit to the corresponding post-processed and locally filtered cryo-EM reconstruction. The resolution at the FSC=0.5 criterion is indicated by a dashed line.

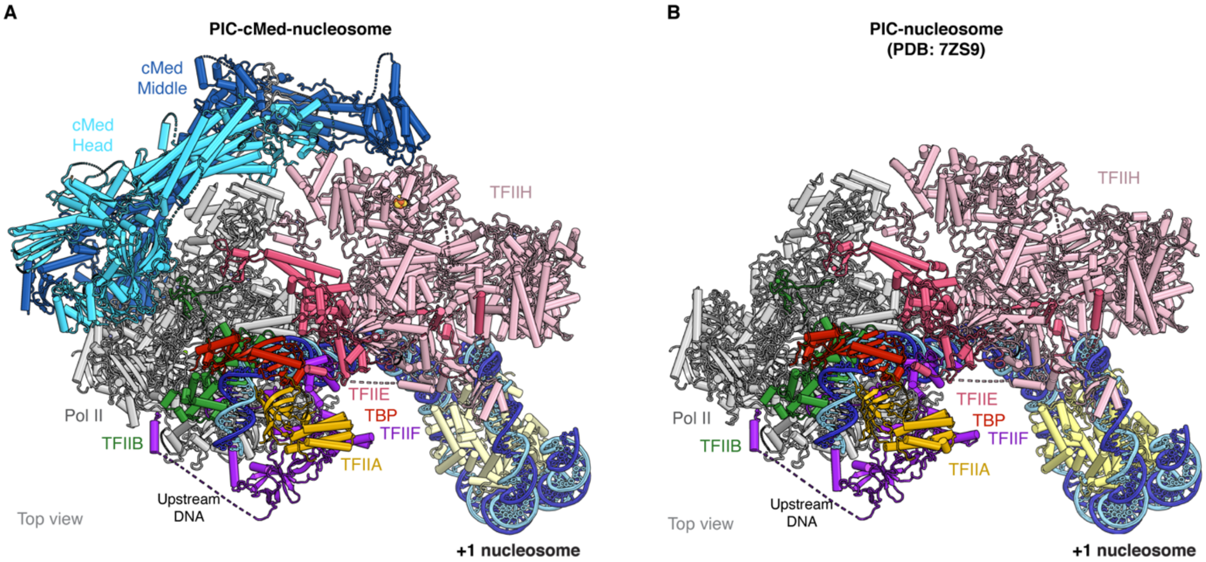

Figure S3. Comparison of PIC-cMed-nucleosome and PIC-nucleosome structures. *(A)* Yeast PIC-cMed-nucleosome complex structure depicted in ribbon representation and viewed from the top. Major submodules are distinguished by color. The DNA template and non-template strands are shown in dark and light blue, respectively. Dashed lines represent flexible linkers. Color code is as in Fig. 1. *(B)* Previously reported yeast PIC-nucleosome complex structure (PDB: 7ZS9) (3) viewed from the top. Major submodules are distinguished by color. The DNA template and non-template strands are shown in dark and light blue, respectively. Dashed lines represent flexible linkers. Color code as in Fig. 1.

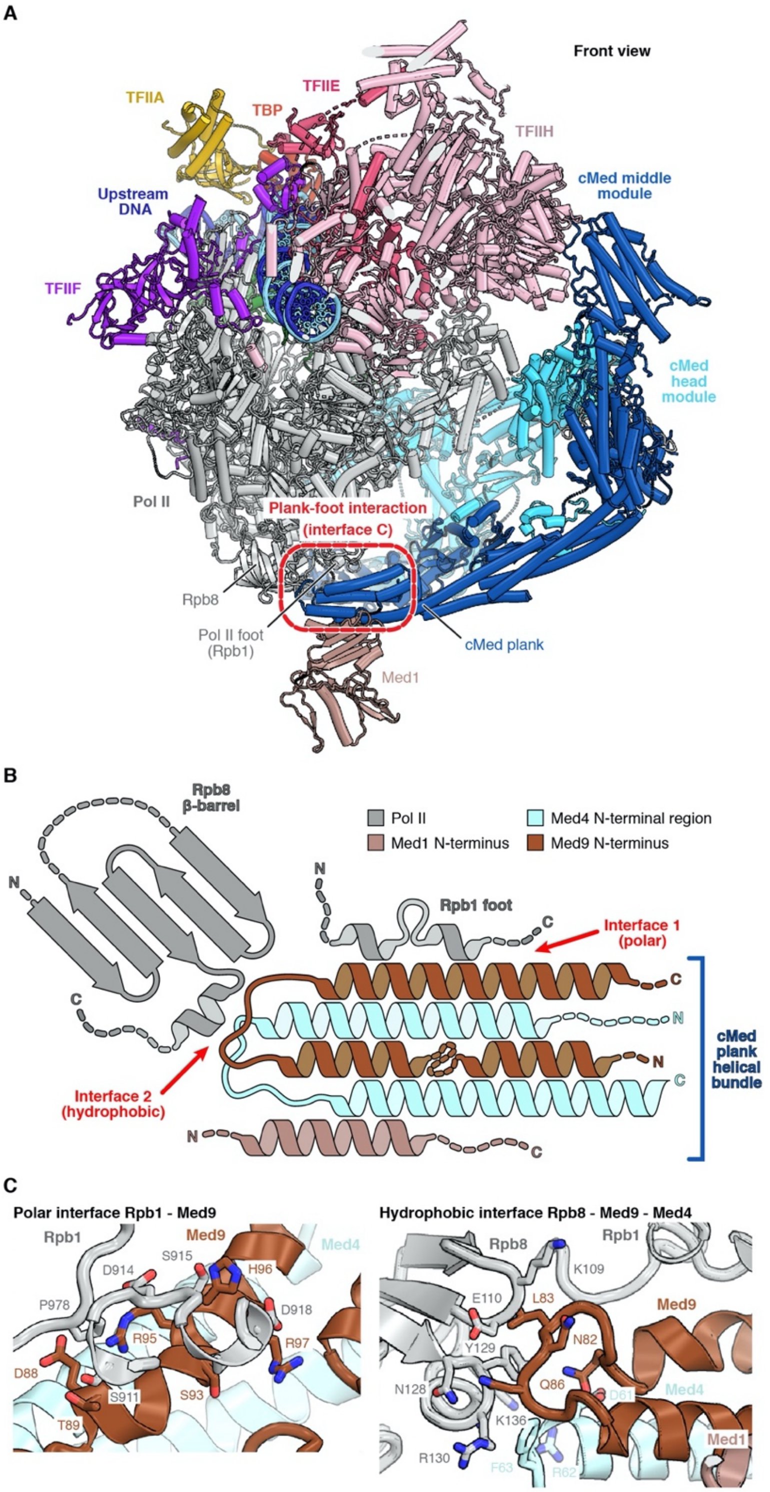

Figure S4. Structural details of the interaction between the 'foot' of Pol II and the cMed plank domain. *(A)* Overview of the yeast PIC-cMed complex viewed from the front. The region of contact between Pol II and the cMed plank (interface C as in (4)) is outlined in red and domains involved in the interaction are indicated. Parts of TFIIH were omitted for clarity. Color code as in Fig. 2. Template and non-template DNA strands are depicted in dark and light blue, respectively. *(B)*

Schematic representation of protein elements involved in cMed-Pol II interface C. Two contacts (marked by red arrows) that are formed between the cMed plank tip and two distinct Pol II subunits contribute to formation of interface C. The interaction between Med9 and the Rpb1 foot domain is dominated by polar residues, whereas Med9, Med4 and the Rpb8 β-barrel constitute a hydrophobic interface that is further stabilized by Med1 (see also *SI Appendix* Fig. S4). Dashed lines represent protein regions that were not modelled or are without relevance for the cMed-Pol II interaction. The color code is indicated. *(C)* Structural details of the contacts between Pol II and cMed. Residues that contribute to formation of the interface and their type of interaction were analyzed by the PISA web server (5) and are represented as sticks with individual labels.

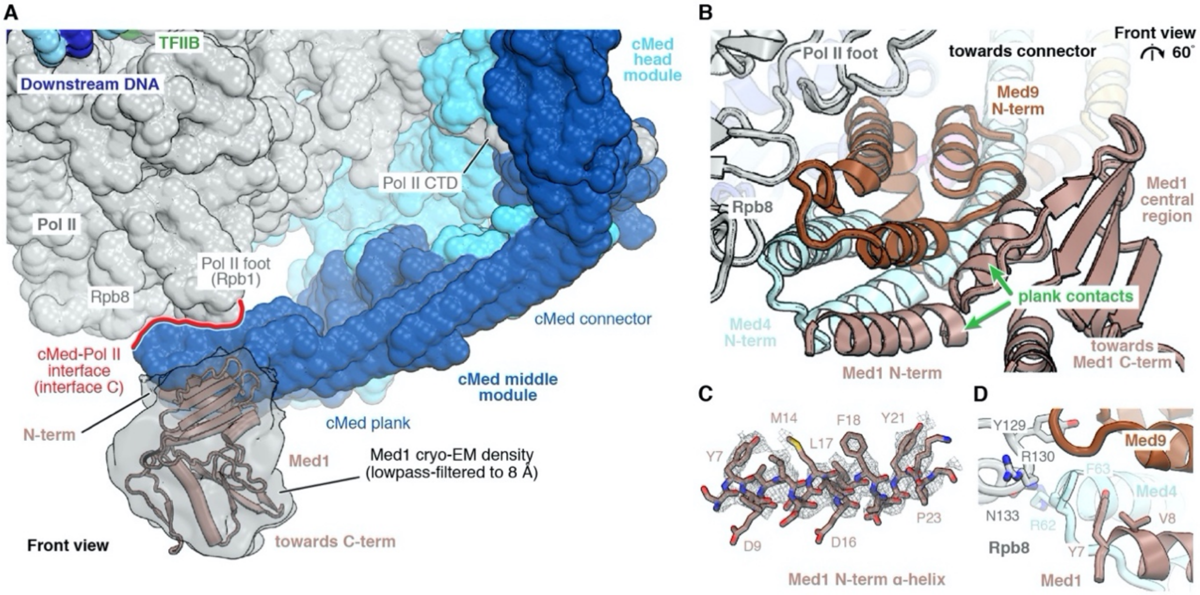

Figure S5. Orientation of Med1 and its interaction with the cMed plank domain. *(A)* Med1 is located in proximity to the cMed-Pol II interface. The surface of the yeast PIC-cMed complex is shown and selected elements are highlighted for orientation. Interface C between Pol II and the cMed plank is demarcated in red. Med1 is depicted in ribbon representation and the lowpass filtered cryo-EM density accounting for the ordered regions of Med1 is provided in translucent dark grey. Flexible regions within Med1 were omitted from the model due to limited structural information *(Methods)*. *(B)* Close-up view of the interaction between the helical bundle at the distal tip of the cMed plank domain and Med1. Two α-helices at the N-terminus of Med1 (demarcated by green arrows) participate in formation of the helical bundle by contacting Med4 and Med9. *(C)* High resolution cryo-EM density permits confident assignment of the Med1 N-terminus. The resolution of the cryo-EM reconstruction (grey mesh) was sufficient to obtain side-chain information for the α-helix at the utmost Med1 N-terminus. Selected bulky residues are labelled for orientation. *(D)* Med1 contributes to the hydrophobic interface between the cMed plank domain and Rpb8. Hydrophobic residues at the front of the N-terminal Med1 α-helix support the Med4-Rpb8 interaction. Contributing residues are depicted as sticks and individually labelled.

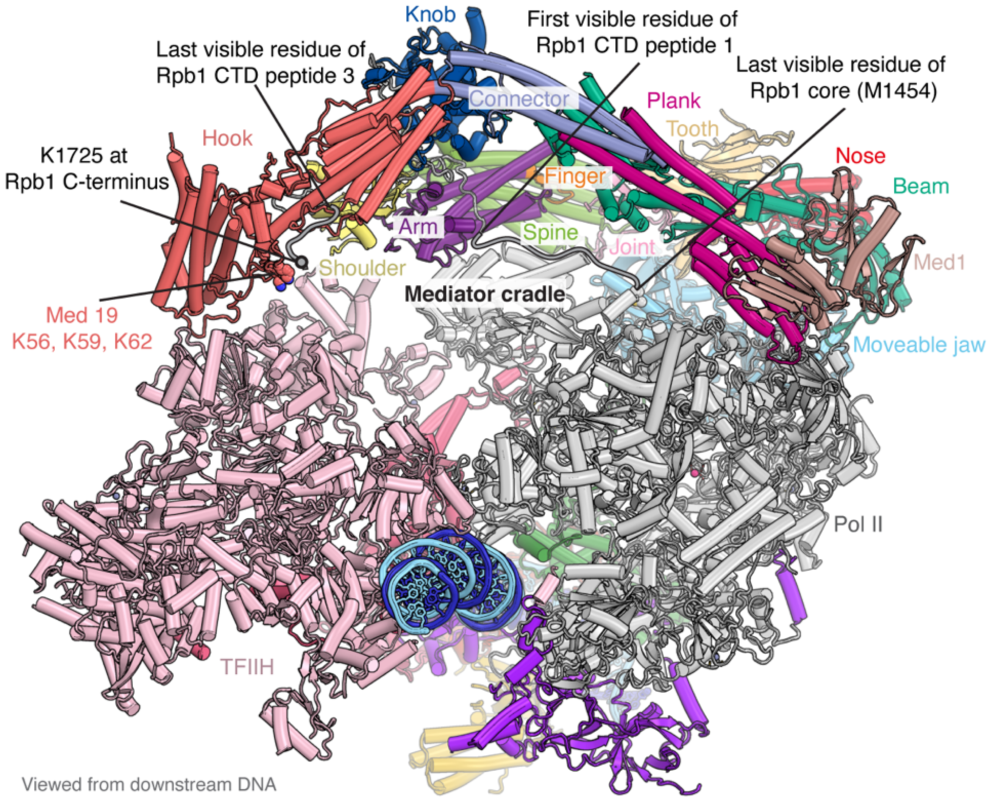

Figure S6. Trajectory of Rpb1 CTD within the PIC-cMed complex. PIC-cMed complex model depicted in ribbon representation and viewed along the exit path of the downstream DNA. The terminal residues of the Rpb1 fragments that are visible in the structure are indicated. Flexible linkers within the CTD between the Rpb1 core and the very C-terminus, which were not observed, are depicted in grey with a black outline. Lysine residue 1725 in the Rpb1 C-terminus which was previously reported to crosslink to lysine residues 56, 59 and 62 of subunit Med19 (6) (shown as spheres) is indicated with a small grey sphere. Color code as in Fig. 1 and 2. Template and non-template DNA strands are depicted in dark and light blue, respectively.

Table S1. Cryo-EM data collection, refinement and validation statistics.

	PIC-cMed complex (EMD-16610) (PDB 8CEN)	PIC-cMed-nucleosome complex (EMD-16611) (PDB 8CEO)
**Data collection and processing**		
Magnification	81,000	81,000
Voltage (kV)	300	300
Electron exposure (e–/Å^2)	42	42
Defocus range (μm)	0.8 to 2.0	0.8 to 2.0
Pixel size (Å)	1.05	1.05
Symmetry imposed	C1	C1
Initial particle images (no.)	5,381,599	5,381,599
Final particle images (no.)	186,599	50,715
Map resolution (Å)	3.0	3.6
FSC threshold	0.143	0.143
Map resolution range (Å)	2.3 – 5.5	2.8 – 7.0
**Refinement**		
Initial models used (PDB code)	7O73, 5OQM	7ZS9, 5OQM
Model resolution (Å)	2.8	3.4
FSC threshold	0.5	0.5
Map sharpening *B* factor (Å^2)	-40	-40
Model composition		
Non-hydrogen atoms	101934	113590
Protein residues	12311	13075
Nucleotides	146	418
Ligands	19	19
B factors (Å^2)		
Protein	109.51	120.54
Nucleotides	145.15	168.23
Ligand	150.14	140.65
R.m.s. deviations		
Bond lengths (Å)	0.004	0.006
Bond angles (°)	0.687	0.652
Validation		
MolProbity score	1.49	1.86
Clashscore	5.82	6.17
Poor rotamers (%)	0.04	0.01
Ramachandran plot		
Favored (%)	96.99	96.74
Allowed (%)	3.00	3.25
Disallowed (%)	0.01	0.01

SI References

1. J. Zivanov *et al.*, New tools for automated high-resolution cryo-EM structure determination in RELION-3. *Elife* **7** (2018).
2. D. Tegunov, P. Cramer, Real-time cryo-electron microscopy data preprocessing with Warp. *Nat Methods* **16**, 1146-1152 (2019).
3. H. Wang, S. Schilbach, M. Ninov, H. Urlaub, P. Cramer, Structures of transcription preinitiation complex engaged with the +1 nucleosome. *Nat Struct Mol Biol* **Online ahead of print.** (2022).
4. S. Schilbach *et al.*, Structures of transcription pre-initiation complex with TFIIH and Mediator. *Nature* **551**, 204-209 (2017).
5. E. Krissinel, K. Henrick, Inference of macromolecular assemblies from crystalline state. *J Mol Biol* **372**, 774-797 (2007).
6. C. Plaschka *et al.*, Architecture of the RNA polymerase II-Mediator core initiation complex. *Nature* **518**, 376-380 (2015).
